# Inhibition of core gene of HCV 3a genotype using synthetic and vector derived siRNAs

**DOI:** 10.1186/1743-422X-7-318

**Published:** 2010-11-13

**Authors:** Saba Khaliq, Shah Jahan, Bushra Ijaz, Waqar Ahmad, Sultan Asad, Asim Pervaiz, Baila Samreen, Mahwish Khan, Sajida Hassan

**Affiliations:** 1 Applied and Functional Genomics Laboratory, National Center of Excellence in Molecular Biology, University of Punjab, Lahore 53700, Pakistan

## Abstract

**Background:**

Hepatitis C virus (HCV) is a major causative agent of liver associated diseases throughout the world, with genotype 3a responsible for most of the cases in Pakistan. Due to the limited efficiency of current therapy, RNA interference (RNAi) a novel regulatory and powerful silencing approach for molecular therapeutics through a sequence-specific RNA degradation process represents an alternative option.

**Results:**

The current study was purposed to assess and explore the possibility of RNAi to silence the HCV-3a Core gene expression, which play complex role in regulation of cell growth and host genes expression essential for infectivity and disease progression. To identify the potent siRNA target sites, 5 small interfering RNAs (siRNAs) against Core gene were designed and *in vitro *transcribed after consensus sequence analysis of different HCV-3a isolates. Antiviral effects of siRNAs showed upto 80% inhibition of Core gene expression by different siRNAs into Huh-7 cells as compared with Mock transfected and control siRNAs treated cells. For long lasting effect of siRNAs, vector based short hairpin siRNAs (shRNAs) were designed and tested against HCV-3a Core which resulted in a similar pattern of inhibition on RNA and protein expression of HCV Core as synthetic siRNAs. Furthermore, the efficacy of cell culture tested siRNA and shRNA, were evaluated for inhibition of HCV replication in HCV infected serum inoculated Huh-7 cells and a significant decrease in HCV viral copy number was observed.

**Conclusions:**

Our results support the possibility of using consensus siRNA and shRNA-based molecular therapy as a promising strategy in effective inhibition of HCV-3a genotype.

## Background

Hepatitis C virus a global public health problem causes a variety of liver-related diseases varying from an asymptomatic condition to hepatocellular carcinoma (HCC). More than 3% of the world's population is chronically infected with HCV especially in developing countries including Pakistan where 6% of population is infected with this viral pathogen [1,2]. The most common HCV genotype in Pakistan is 3a followed by 3b and 1a with a strong correlation between chronic HCV infection (genotype 3a) and HCC in Pakistan [3-5]. In most of the cases HCV escapes immune system while the standard treatment for HCV, a combination therapy of pegylated interferon α (PEG-IFN-α) and guanosine analog ribavirin, has limited efficiency, significant expense, poor tolerability and assure long term eradication of the virus in 54-56% treated patients [6-8]. Therefore, development of molecular approaches like RNA interference, a sequence specific gene silencing mechanism which has found to work in mammalian cells, is needed against HCV. RNAi can be introduced into the cells using two different approaches: (i) chemically synthesized 21-23nt small interfering RNAs (ii) a 80-100nt short hairpin RNA (shRNA) expression cassettes which is then processed into active siRNA by the host [9,10]. Both siRNA and shRNA induce post-transcriptional gene silencing into mammalian cells in the same manner without activating an interferon response [11]. Based on these finding a number of investigators have examined antiviral effects of siRNAs against a number of candidate genes of different diseases that interfere with replication of animal viruses.

HCV is highly susceptible to RNAi as replication occurs in the cytoplasm of liver cells, destruction of HCV RNA could induce failure of HCV replication. Recent experiments with HCV subgenomic and genomic replicon systems show that HCV replication is sensitive to RNAi activity [12-19]. HCV Core located at the N-terminus of the polyprotein is the viral nucleocapsid protein that packages the viral RNA in interaction with the envelope proteins (E1 and E2) [20-22]. Core can be separated into two domains: an N-terminal two third hydrophilic domain (D1) and a C-terminal one third hydrophobic domain (D2) [23]. The D1 domain of Core protein has RNA-binding and homo-oligomerization property forming the viral nucleocapsid with numerous functional activities. The D2 domain is required for proper folding of domain D1 and membrane characteristics of the Core [24,25]. Core is a multifunctional protein influencing a whole array of host cell functions, including apoptosis, HCV associated-steatosis, immune cell functions, cell transformation, signal transduction, and transcriptional regulation leading to HCC [26-32]. A relationship between substitutions in Core region of HCV-3a with enhanced insulin resistance and oxidative stress has been observed. Moreover, HCV induced-steatosis is more frequent and severe in HCV genotype 3 patients due to the presence of specific steatogenic sequences within this genotype [33-39]. Since Core plays crucial roles in HCV infection and immunity, it is helpful to use RNAi against it by targeting virion formation as new therapeutic option.

In the present study, we aimed to compare the effect of siRNA and shRNA to specifically target Core gene of local HCV-3a genotype as new options for developing a rational antiviral strategy. To avoid potential escape mutants, as the target sequence we selected HCV Core gene whose sequence conservation is extremely high among different HCV genes. It is expected that cleavage of Core protein mRNA will inhibit nuclear transport and virus replication. We report here that RNAi targeted against Core effectively inhibited Core gene RNA and protein expression in a dose dependant manner in Huh-7 cells irrespective of mode of delivery. The present study demonstrates that the RNAi-mediated silencing of the HCV-3a Core gene may be one of the important therapeutic opportunities against HCV-3a genotype.

## Results

### Efficient siRNAs targeting HCV-3a Core gene screening

**Figure 1:**
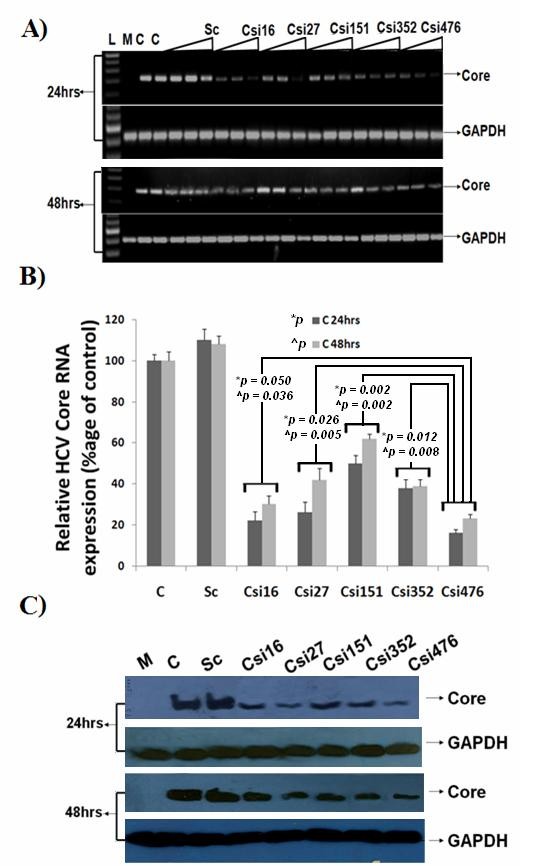
**HCV -3a Core specific siRNAs inhibit Core expression**. **A) **Dose dependent silencing effect of synthetic siRNA against Core gene of HCV-3a. Huh-7 cells were transfected with 0.4 μg of constructed HCV Core vector or Mock along with or without 10, 20 and 40 nM of siRNAs for 24 and 48 hrs. Cells were harvested and relative RNA determinations were carried out using semi-quantitative RT-PCR. Gene expression results are given for increasing concentrations of Csi16, Csi27, Csi151, Csi352, and Csi476 siRNAs against HCV-3a Core. Expression levels for Mock-transfected (M), HCV-3a Core expression plasmid (C), scramble siRNA (Sc), 100 bp DNA Ladder (L) and GAPDH are also shown. **B) **Quantitative Real Time PCR analysis of Core 3a or Mock-treated Huh-7 cells along with or without 40 nM of siRNAs for 24 and 48 hrs in comparison to Mock. Gene expression results from Real Time PCR shows that Csi16, Csi352, and Csi476 siRNAs against HCV-3a Core decrease RNA expression after 24 and 48 hrs transfection. GAPDH was used as internal control. Each independent experiment was performed having triplicate samples. The *p *values indicate significant differences between the connected groups. Error bars indicate mean S.D, Csi476 verses other siRNA: p* for 24 and *p*^ for 48 hrs **C)**. Silencing of HCV-3a Core gene by siRNAs using specific antibodies showed reduction at protein expression level. The protein expression levels were determined by western blot analysis after 24 and 48 hrs transfection with Mock (M), HCV-3a Core expression plasmid (C) with and without HCV-3a siRNAs (Csi16, Csi27, Csi151, Csi352, and Csi476) and scramble siRNA (Sc) in Huh-7 cells.

siRNA directed against HCV are expected to successfully block replication cycle since HCV being a RNA virus replicates in the cytoplasm of liver cells without integration into the host genome. To efficiently silence Core gene expression by in-vitro transcribed siRNAs and avoid sequence variants, the most conserved target sequence were chosen after analyzing a consensus sequence of local HCV-3a Core sequences and reference sequences retrieved from GenBank. Negative control siRNA (scrambled siRNA) with the same nucleotide composition as the experimental siRNA which lacks significant sequence homology to the HCV and human genome was designed (Table 1). siRNAs were transfected with pCR3.1/FlagTAG/Core vector into Huh-7 cells to investigate their specificity and expression levels both at mRNA and protein level via semi-quantitative RT-PCR, Real Time PCR and western blotting. To assess the effects of chemically synthesized siRNAs on HCV-3a Core, increasing concentration of siRNAs (Csi16, Csi27, Csi151, Csi352 and Csi476) were introduced into the cells for 24 and 48hrs. All inhibited HCV Core RNA in a dose-dependent manner (10, 20 and 40 nM) examined by semi-quantitative RT-PCR. The inhibitory effect of siRNAs Csi16, Csi352 and Csi476 are stronger even at low concentration, while Csi27 and Csi151 showed more effect after 24 hrs transfection at 40 nM than at 48 hrs post transfection as compared to scrambled siRNA (Figure 1A). These results were further confirmed by Real Time PCR, using primer specific to HCV Core and GAPDH. Based on the relative study, the percentages of HCV mRNA in siRNA (40 nM) co-transfected cells over scramble was calculated, normalizing it with GAPDH. The results of relative quantitative analysis revealed that the mRNA level of HCV Core was decreased to 78% in cells treated with Csi16, 74% with Csi27, 50% with Csi151, 62% with Csi352 and 84% with Csi476 at 24 hrs post transfection while 70% Csi16, 58% with Csi27, 38% with Csi151, 61% with Csi352 and 77% with Csi476 decreased 48 hrs post transfection. The most effective siRNA reaching a maximum inhibition of 60-80% after 24 and 48 hrs transfection were Csi16, Csi27, Csi352 and Csi476 (Figure 1B). Western blot analysis of protein extracts derived from the siRNA transfected cells showed that HCV Core protein expression was reduced in cells co-transfected with Core specific siRNA, but not in the cells transfected with scrambled siRNA 24 and 48 hrs post-transfection. The Csi476 siRNAs was found to be more effective with upto 70% decreased protein expression after 24 hrs while all siRNA inhibited protein expression upto 50-65% after 48 hrs transfection (Figure 1C). These data suggest that synthetic Core siRNA not only has a negative effect on Core mRNA but also it could decrease viral protein production.

**Table 1:** Sequence of siRNA oligonucleotides directed against Core gene of HCV 3a genotype

**No**.	siRNA name	Sequences 5'-3'
1	Csi16-antisense	AAACCTCAAAGAAAAACCAAACCTGTCTC
2	Csi16-sense	AATTTGGTTTTTCTTTGAGGTCCTGTCTC
3	Csi27-antisense	AAACCAAAAGAAACACCATCCCCTGTCTC
4	Csi27-sense	AAGGATGGTGTTTCTTTTGGTCCTGTCTC
5	Csi151-antisense	AAAACTTCTGAACGGTCACAGCCTGTCTC
6	Csi151-sense	AACTGTGACCGTTCAGAAGTTCCTGTCTC
7	Csi352-antisense	AATTTGGGTAAAGTCATCGATCCTGTCTC
8	Csi352-sense	AAATCGATGACTTTACCCAAACCTGTCTC
9	Csi476-antisense	AAGACGGGATAAATTTCGCAACCTGTCTC
10	Csi476-sense	AATTGCGAAATTTATCCCGTCCCTGTCTC

### Inhibition of HCV Core expression in HCV serum infected Huh-7 Cells by siRNA

**Figure 2:**
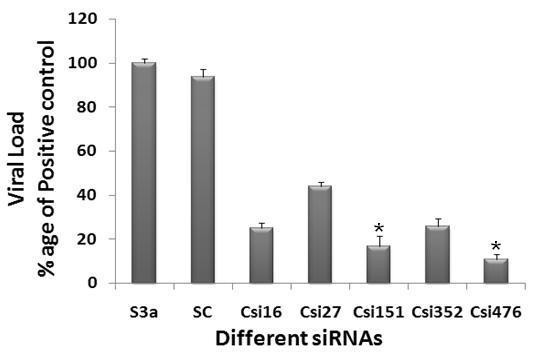
**Silencing effect of HCV-3a genes-specific siRNAs show a dramatic reduction of viral titer in Huh-7 cells infected with HCV-3a sera**. Huh-7 cells were infected with high titer sera samples from HCV-3a patients (S3a) to establish in vitro cell culture model of HCV-3a, cells were maintained overnight at 37°C in 5% CO_2 _for three days. Cells were harvested after siRNA treatment 48 hrs post transfection and intracellular HCV RNA levels were quantified by Real Time PCR. Data is expressed as mean percent viral load of non-siRNA treated samples. Nine independent experiments each with triplicate determinations were performed with different sera infected cells. Error bars indicate, mean S.D p < 0.05 verses S3a.

Since HCV replication in cell culture is limited to human hepatocytes and their derivatives, now several reports have verified that HCV can replicate in Huh-7 cells through detection of viral genes as well as viral copy number by Real Time PCR in both cells and supernatant [40-43]. The present study was undertaken to design and test siRNA as an alternative therapy against HCV, the results indicate that siRNA targeting HCV-3a Core has the ability to inhibit mRNA and protein in Huh-7 cells. We speculated that Core siRNAs in Huh-7 cells has the ability to inhibit the replication of HCV. To test this possibility, Huh-7 serum infected cells were treated with the Core synthetic siRNAs and subsequently incubated for 3 days. Real Time PCR was performed with HCV specific primers (5'UTR) to analyze the down regulation of RNA by Core specific siRNAs. Maximal inhibition (70-80%) of HCV transcript levels was detected on day 3 post-transfection in HCV serum infected Huh-7 cells. No significant inhibition was detected in cells transfected with the negative control siRNA. This result was in accordance with Zekri et al. 2009 [41] who also showed best inhibitory effect of siRNAs against 5'UTR on 3^rd ^day of post-transfection. siRNAs against Core gene showed a dramatic reduction in HCV viral RNA, Csi476 showed a maximum inhibition of about 89%, while Csi352, Csi16 and Csi27 showed 83%, 68%, and 62% inhibition respectively. Csi151 being the least effective siRNA showed only 54% inhibition in viral load (Figure 2). Together, these data suggest a negative impact of chemically synthesized Core siRNA on HCV replication that could be used for the down regulation of Core expression for preventing HCV pathogenesis.

### Targeting HCV Core gene via plasmid-based siRNA expression system

**Figure 3:**
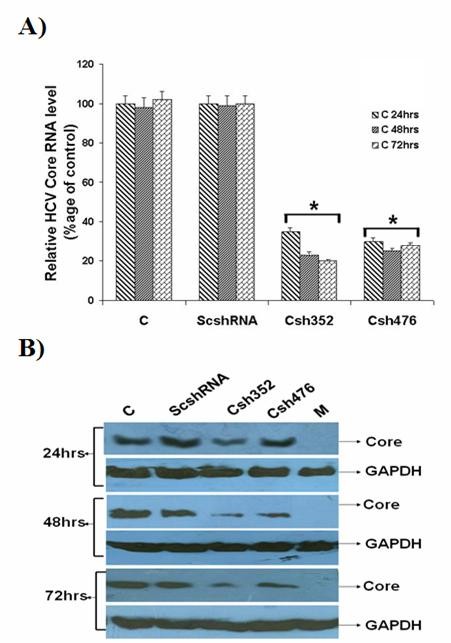
**HCV-3a Core specific shRNAs inhibit mRNA expression**. **A) **Total cellular RNA extracted from transfected Huh-7 after 24, 48 and 72 hrs post-transfection. Gene expression results from Real Time PCR showed that Csh352 and Csh476 shRNAs against HCV-3a Core decrease RNA expression using gene specific primers in comparison to Mock with GAPDH as internal control. Three independent experiments were performed having triplicate samples. Error bars indicate mean S.D, *p < 0.01. **B) **Silencing of HCV-3a Core gene by shRNAs using specific antibodies showed reduction at protein expression level determined by western blot analysis after 24, 48 and 72 hrs transfection with Mock (M), HCV-3a Core expression plasmid (C) with and without HCV-3a shRNAs (Csh352 and Csh476) and scramble shRNA (ScshRNA) in Huh-7 cells. Protein levels for GAPDH gene are also shown as internal control.

The half life of synthetic siRNA duplexes is too short and in some conditions may not stay long enough for complete elimination of virus in infected cells. Vector based intracellular delivery of siRNA offers an alternative strategy which allows continuous production of siRNA within the cells and permits long-term eradication of viral gene expression [9]. Therefore, we proposed that plasmid based vector offers an alternative for intracellular delivery of siRNA for complete inhibition of viral gene expression and replication. To determine this possibility, the activity of two siRNA expression vectors (shRNAs) designed after screening of Core specific siRNAs, which inhibited Core expression at least 70%, were determined (Table 2). Two annealed shRNA oligonucleotides (Csh352 and Csh476) were cloned into pUbCeGFP vector containing the shRNA expression cassettes, under the control of UbC promoter. The specific inhibitory effect of shRNA against HCV-3a Core was determined with and without control scramble siRNA vector (ScshRNA). Quantitative Real Time PCR was used to investigate whether intracellular expression of shRNA inhibited HCV Core RNA expression levels in Huh-7 cells showing 70-75% with Csh352 and 75-80% with Csh476, while no inhibitory effect was detected in cells transfected with scramble siRNA even after 72 hrs of transfection. Our preliminary results have shown that the shRNA inhibited HCV-3a Core RNA expression to almost the same extent as the synthetic siRNAs (Figure 3A). The specific inhibitory effects of shRNAs against HCV-3a Core protein levels were also determined using western blotting. Both shRNAs effectively inhibited HCV Core protein expression to 85% (Csh352) and 80% (Csh476) even after 72 hrs of transfection as compared to either Mock or scrambled shRNA transfected cells with no effect on expression levels of GAPDH gene, suggesting that the suppressive effects of shRNA against Core were directed specifically to the HCV gene (Figure 3B).

**Table 2:** Sequence of shRNAs oligonucleotides used in the study

No	shRNA name	Sequences 5'-3'
1	ScshRNA- sense	CTGCTGTTGACAGTGAGCGAAAGTCGAGTCGCGTATGCAGGGTGAAGCCACAGATGAACCTGCATACGCGACTCGACCTGCCTACTGCCTCGGACTTCAAGGG
2	ScshRNA- antisense	AATTCCCTTGAAGTCCGAGGCAGTAGGCAGGTCGAGTCGCGTATGCAGGTTCATCTGTGGCTTCACTGCATACGCGACTCGACTTTCGCTCACTGTCAACAGCAGGTAC
3	Csh352- sense	CTGCTGTTGACAGTGAGCGAAAATCGATGACTTTACCCAAAGTGAAGCCACAGATGAATTTGGGTAAAGTCATCGATCTGCCTACTGCCTCGGACTTCAAGGG
4	Csh352- antisense	AATTCCCTTGAAGTCCGAGGCAGTAGGCAGATCGATGACTTTACCCAAATTCATCTGTGGCTTCACTGGGTAAAGTCATCGATTTTCGCTCACTGTCAACAGCAGGTAC
5	Csh476-sense	CTGCTGTTGACAGTGAGCGAAATTGCGAAATTTATCCCGTCGTGAAGCCACAGATGAAGACGGGATAAATTTCGCAACTGCCTACTGCCTCGGACTTCAAGGG
6	Csh476-antisense	AATTCCCTTGAAGTCCGAGGCAGTAGGCAGTTGCGAAATTTATCCCGTCTTCATCTGTGGCTTCACCGGGATAAATTTCGCAATTTCGCTCACTGTCAACAGCAGTAC

### Inhibition of HCV Core expression in HCV serum infected Huh-7 cells by shRNA

**Figure 4:**
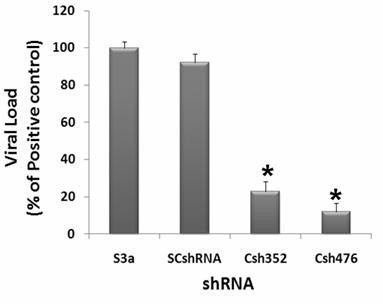
**Silencing effect of HCV-3a Core-specific shRNAs show a dramatic reduction of viral titer in Huh-7 cells infected with HCV-3a sera**. Huh-7 cells were infected with high titer sera samples from HCV-3a patients to establish in vitro cell culture model of HCV-3a, cells were maintained overnight at 37°C in 5% CO_2_, incubation was continued for 48 hrs. Huh-7 infected cells were again plated and transfected with shRNAs against HCV-3a genes for additional 48 hrs. Cells were harvested and intracellular HCV RNA levels were quantified by Real Time PCR. Data are expressed as mean percent viral load of non-siRNA treated samples. Nine independent experiments with different sera infected cells and each with triplicate samples were performed. Error bars indicate, mean S.D p < 0.01 verses S3a.

We speculated that Core specific shRNA in Huh-7 cells has the ability to inhibit the replication of HCV similar to chemically synthesized siRNAs. To test this possibility of shRNA on HCV RNA replication, Huh-7 serum infected cells were treated with the Core shRNAs and subsequently incubated for 3 days. Real Time PCR was performed with HCV specific primers to analyze the down regulation of viral RNA by Core specific shRNAs and an approximate 90% decrease in HCV RNA levels incubated with Csh476 while Csh352 showed 80% decrease in HCV-serum infected Huh-7 cells. No significant inhibition was detected in cells transfected with the control shRNA (ScshRNA) (Figure 4). Taken together, these results indicated that in serum-infected Huh-7 cells, direct transfection of shRNAs can specifically produce RNAi against HCV and reduce HCV viral titer. Therefore, the use of selective gene silencing like chemically synthesized and vector based siRNA for the down regulation of HCV genes might be a target for preventing HCV induced HCC development.

## Discussion

HCV is a major cause of chronic hepatitis in Pakistan with genotype 3a being the most prevalent type [3,4]. Conventional therapies for treating HCV have their limitation and alternative anti-HCV strategies are urgently needed. As a gene silencing mechanism, RNAi represents an exciting technology with potential applications for treatment of viral diseases and investigation of gene functions. A potential problem that may arise in RNAi based approach is the error prone nature of HCV genome with generation of quasispecies during chronic HCV infection but this problem can be overcome by designing siRNAs against highly conserved region of HCV. The region encoding Core is well conserved; results of nucleotide and deduced amino acid sequence analysis across diverse strains of HCV reveal 81-88% nucleotide and 96% amino acid sequence homology [44,45]. During the course of present study 92% nucleotide sequence homology was observed between different HCV-3a Core isolates. In the current study, we were able to show that the introduction of synthetic and vector based siRNAs into target cells containing HCV Core caused a dramatic decrease of viral RNA and protein expression.

HCV infects liver cells, replicates efficiently and continuously in liver derived Huh-7 cells [46]. Huh-7 cells are most widely used for liver associated diseases and fundamental studies for the development of antiviral agents against HCV as infectious cell culture system [47-49]. Liu et al. 2006 [14] and Kim et al., 2006 [50], has designed siRNA against HCV 1b and 1a genome to explore the silencing of structural genes and showed significantly less expression in a dose-dependent manner. Specificity of binding to the target RNA and functional importance of the targeted region is essential for effective gene silencing and successful antiviral activity. As different domains of Core have different functions, N-terminal 1-50 amino acid contains RNA and DNA binding domain, nuclear localization signals (NSL) while C-terminal 91-191 aa inactivates and binds to Leucine zipper and 160-194 aa to apolipoprotein II [28,51], siRNAs were designed against each domain. Moreover, N-terminal of Core induces apoptosis and necrosis higher than those of C-terminal while middle domain with lowest induced apoptosis and necrosis percentages [52]. The results in this study demonstrated that siRNAs directed against domains (N-terminal and C-terminal) of HCV-3a Core gene resulted in specific inhibition of HCV RNA synthesis (60-80%), whereas the control siRNA did not affect HCV and GAPDH mRNA level. To determine whether the synthesized siRNA could effectively silence target protein expression, we used Western blotting to detect the expression of Core protein in transfected cells. Csi476 was the most effective (upto 70%) siRNA among all siRNAs which were designed against C and N-terminal of Core gene making it a potent site for HCV-3a Core inhibition (Figure 1). Liu et al., 2006 [14] showed the effect of different siRNAs directed against HCV 1b Core region (28-509 nt) but the effect of 2 siRNAs ranging from 28-200 nt were found to be most effective upto 70% while the effect of others are stated as in effective. Contrary to these results, in the present study, we found C- and N-terminal siRNAs to be all effective upto 70-80% with Csi151 as least effective. Two siRNAs against HCV genotype 1b used by Liu et al. 2006 has almost the same sequence as used in the present study, Csi27 of our study also showed the same level of inhibition upto 70% as by CsiRNA 1 used by Liu et al. The Csi352 also shares sequence homology to CsiRNA 3 but the difference in siRNA effect cannot be explain completely as the level of inhibition effect of CsiRNA 3 is not stated. The difference in siRNAs effect may be due to base pair differences between genotype 1b and 3a as these differences has also been found to be involved in several pathogenic disease progressions [35,36].

Recently different groups have studied the HCV replication in serum infected liver cell lines which mimics the naturally occurring HCV virions biology and kinetics of HCV infection in human. We infected Huh-7 cells with native viral particles from HCV-3a positive serum using the same protocol as established [40-43,53]. HCV-3a Core siRNAs used in the present study was further screened against HCV serum infected Huh-7 cells. An exciting finding of this study is decline of HCV viral titer to a maximum of 90% with gene specific siRNAs. HCV replication in the Huh-7 cells was observed through detection of 5'UTR of viral copies by Real Time PCR in cells 3^rd ^day post infection. HCV-3a Core siRNAs showed a range 54-90% inhibition in viral titer with Csi476 showing upto 89% and Csi151 only 54% inhibition (Figure 2). Our data is in agreement with Zekri et al., 2009 [41], who demonstrated that siRNAs against 5'UTR of HCV genotype-4 inhibited HCV replication in serum infected Huh-7 cells. Bian et al., 2009 [54] reported that 14 amino acids from the C-terminus of Core gene are required for proper function of E1 and at least 12 amino acids from C-terminus of E1 genes are required for E2 function, influencing the proper glycosylation of E1 and E2 gene. Effect of HCV-3a Core siRNA on HCV viral titer reduction is possibly due to the interaction between different HCV regions and may also due to the simultaneous degradation of HCV genomic RNA (as HCV genome contains a positive sense ssRNA). Treatment of siRNAs revealed significant inhibitory effects on HCV copy number, indicating that siRNAs might be an efficient strategy for molecular HCV therapeutics.

Various strategies have been adopted in delivery options of RNA to cells, either directly or through the introduction of expression vectors in which vector based strategies are foremost as efficient delivery of siRNA is major hindrance in effective silencing of HCV replication. Unexpectedly, siRNAs directed against the 5'UTR of HCV genotype 1b had less effect on HCV replication while the coding regions (particularly the highly conserved protein Core) are more feasible target for RNAi mediated gene silencing [50]. The expression of shRNAs targeting specific portions of HCV Core protein expressing in Huh-7 cells was studied showing down regulation of Core protein [55,56]. In both studies shRNAs directed against C-terminal of Core region showed inhibition of HCV 1b Core upto 70%. Keeping in view these observations, the expression of shRNAs targeting specific portions of HCV-3a Core protein is a critical factor for effective silencing. C-terminal region of Core was selected for shRNA as biophysical characterization of the Core protein indicates that residues 125-179 are critical for the proper folding and oligomerization of the Core protein [23]. A mammalian expression vector that directed the synthesis of fully processed siRNAs (Csi352 and Csi476) in HCV-3a Core transfected Huh-7 cells was used. To confirm the inhibitory effects of shRNA in the cells, Real Time PCR and Western blotting was performed after 24, 48 and 72 hrs post transfection. These shRNAs suppressed the expression of Core mRNA determined by Real Time PCR. This inhibition was not shRNA side effect as GAPDH mRNA levels were comparable both with control and Mock treated cells. Both siRNA expression plasmids, Csh352 and Csh476, displayed potent gene silencing effects (upto 75-80%) on HCV Core protein expression and viral RNA. Furthermore, transfection of DNA-based vectors expressing siRNAs (shRNAs) was as effective as that of synthetic siRNA in suppressing HCV RNA (Figure 3). In serum infected Huh-7 cells, shRNA Csh476 was more effective upto 90% inhibition in HCV viral titer than Csh352 which showed 80% inhibition (Figure 4). Present study provides experimental evidence that sequence specific degradation mediated by shRNA expression in the Core expressing Huh-7 cells down regulates HCV-3a Core protein expression.

Our results demonstrate that careful and consensus based sequence selection of targets for siRNA is mandatory, not only to achieve maximum effectiveness, but may also be able to avoid adverse side-effects for therapeutic applications. Based on the experiments performed in this study, it can be concluded that siRNAs directed against specific domains were more efficient in silencing viral gene expression. Furthermore, data presented in this study, also suggest that siRNA targeting HCV-3a Core can elicit viral RNA from infected cell and potentially offer an efficient therapeutic option for HCV infection. These results are in agreement with the previous studies, suggested that siRNA is the most efficient nucleic acid based antiviral approach that can be utilized to degrade HCV genome in the infected cells in a genome sequence specific manner. In conclusion the efficiency of our siRNA in inhibiting HCV-3a replication in cells suggests that RNAi (synthetic or vector based siRNA) may play a role in clearance of virus during HCV-3a infection.

## Methods

### Source of samples

The local HCV-3a patient's serum samples used in this investigation were obtained from the CAMB (Center for Applied Molecular Biology) diagnostic laboratory, Lahore, Pakistan. Serum samples were stored at -80°C prior to RNA extraction for cloning and viral inoculation experiments. Quantification and genotype was assessed by CAMB diagnostic laboratory, Lahore, Pakistan. Patient's written consent and approval for this study was obtained from institutional ethics committee.

### Plasmid construction

For the construction of expression plasmid, viral RNA was isolated from 100 μl serum aliquots using Gentra RNA isolation kit (Gentra System Pennsylvania, USA) according to the manufacturer's instructions. 100-200 ng extracted viral RNA was used for RT-PCR using the SuperScript III one-step RT-PCR system (Invitrogen Life technologies, USA). HCV complementary DNA (cDNA) encoding the full length Core protein (amino acid 1-191 of HCV-3a) were amplified employing forward primer 5'GCGATATCATGAGCACACTTCCTAAA'3 and reverse primer 5'AATCTAGATCATGGCTGCTGGATGAAT'3. PCR products were cloned into pCR3.1 mammalian expression plasmid (kindly provided by Dr. Zafar Nawaz, University of Miami, USA) with FlagTAG inserted at the 5' end of the Core gene with *EcoRV *and *XbaI *restriction sites.

### Synthesis of siRNA and shRNA expression vectors

We adopted two methods, siRNA and shRNA mediated expression, to express RNAi mechanism against Core region of HCV-3a genome. siRNA oligonucleotides were designed to the most conserved target regions using the Ambion's siRNA design tool http://www.ambion.com/techlib/misc/siRNA_finder.html. After sequencing of local HCV-3a patient's serum samples (GenBank accession numbers: FJ009580-FJ009586, EU266534-EU266536) from DNA sequencing facility at CAMB, Lahore, Pakistan and Full length HCV-3a reference sequences obtained from GenBank (accession numbers: D17763, AF046866, D28917 and several partial sequences). These sequences were aligned by using the free software (CLUSTAL_W option of MEGA v.3.1). The designed siRNAs (HCV-3a Core and control Scrambled) were synthesized using Silencer siRNA construction kit according to the manufacturer's instruction (Ambion, USA).

To generate gene specific siRNA expression plasmids (pUbC-shRNAs), two effective siRNA target regions at 3' end of Core gene were selected. Sense and antisense strands of shRNA oligonucleotides were chemically synthesized, annealed at 95°C for 3 min followed by slow cooling and then cloned into the pUbCeGFP plasmid (provided by Dr. Zafar Nawaz, University of Miami, USA) containing UbC promotor. Scrambled shRNA (control) cloned into the same vector was used as negative control in all experiments.

### Cell culture and transfection

Huh-7 cell line was kindly provided by Dr. Zafar Nawaz (University of Miami, USA) and maintained in Dulbecco's modified eagle medium (DMEM) supplemented with 100 μg/ml penicillin; streptomycin and 10% fetal bovine serum referred as complete medium (Sigma Aldrich, USA) at 37°C with 5% CO_2_. The medium was renewed every 3rd day and passaged every 4-5 days. Viable cells were counted using 0.5% trypan blue (Sigma Aldrich, USA).

The cells were transfected with Core specific (Csi16, Csi27, Csi151, Csi352, and Csi476) or scrambled siRNAs along with HCV-3a Core vector (0.4 μg of constructed vector) to analyze inhibition of HCV-3a Core siRNAs. Briefly, cells were seeded in 24-well (1 × 10^5^/well) or 6-well (5 × 10^5^/well) plates and cultured in complete medium until they became 60-80% confluent. Cells in 24-well plates were transiently transfected with 10, 20, 40 nM/well of specific siRNAs or scrambled siRNA (Sc) along with 0.4 μg of HCV-3a Core in serum free media using Lipofectamine™ 2000 (Invitrogen Life technologies, CA) according to the manufacturer's protocol. After 6 hrs incubation at 37°C in 5% CO_2_, complete medium was added to the cells. Protein analysis was carried out for above mentioned experiments in 6-well plates with 100 nM/well of each siRNA. Cells were harvested at 24 hrs post-transfection for gene expression analysis. For assessing the silencing effect of plasmid mediated siRNAs on HCV-3a Core protein, pUbC-shRNA expression vectors namely Csh352 and Csh476 were co-transfected (3 μg in 6-well plate) with HCV-3a Core (1 μg) expression plasmids using Lipofectamine™ 2000 as described above.

### Isolation of RNA and Gene expression analysis

Total RNA from transfected and non-transfected cells was isolated using TRIzol reagent (Invitrogen life technologies, CA), 24 and 48 hrs post-transfection. To analyze the effect of siRNA and shRNA on Core gene, cDNA was synthesized with 1 μg of total RNA, using Superscript III cDNA synthesis kit (Invitrogen life technologies, CA) and semi-quantitative RT-PCR was performed using primers of Core gene and GAPDH as control. Quantitative Real Time PCR was carried out using Real Time ABI 7500 system (Applied Biosystems Inc, USA) with SYBR Green mix (Fermentas International Inc, Canada) using gene specific primers: Core 3a forward primer 5'GGACGACGATGACAAGGACT'3 and Core 3a reverse 5'GGCTGTGACCGTTCAGAAGT'3. GAPDH gene was used for normalization as control using forward primer 5'ACCACAGTCCATGCCATCAC'3 and reverse primer 5'TCCACCACCCTGTTGCTGTA'3. The relative gene expression analysis was carried out by the SDS 3.1 software (Applied Biosystems Inc, USA). Each individual experiment was performed in triplicate.

### Western blotting

To determine the protein expression levels of HCV Core, the transfected [with and without HCV-3a siRNAs (Csi16, Csi27, Csi151, Csi352, and Csi476 and scramble siRNA) and with and without HCV-3a shRNA transfected cells (Csh352, Csh476 and scramble shRNAs expression vector)] and non-transfected cells were lysed with ProteoJET mammalian cell lysis reagent (Fermentas, Canada). Equal amounts of total protein were subjected to electrophoresis on 12% SDS-PAGE and electrophoretically transferred to a nitrocellulose membrane following the manufacturer's protocol (Bio-Rad, CA). After blocking non-specific binding sites with 5% skimmed milk, blots were incubated with primary monoclonal antibodies specific to HCV Core and GAPDH (Santa Cruz Biotechnology Inc, USA) and secondary Horseradish peroxidase-conjugated anti-goat anti-mouse antibody (Sigma Aldrich, USA). The protein expressions were evaluated using chemiluminescence's detection kit (Sigma Aldrich, USA).

### Viral inoculation and co-transfection with siRNA

Huh-7 cell line was used to establish the in vitro replication of HCV. A similar protocol was used for viral inoculation as established by Zekari et al. 2009 [41] and El-Awardy et al. 2006 [42]. High viral titer > 1 × 10^8 ^IU/ml from HCV-3a patient's was used as principle inoculum in these experiments. Huh-7 cells were maintained in 6-well culture plates to semi-confluence, washed twice with serum-free medium, then inoculated with 500 μl (5 × 10^7^IU/well) viral load of HCV-3a sera and 500 μl serum free media. Cells were maintained overnight at 37°C in 5% CO_2_. Next day, adherent cells were washed three times with 1× PBS, complete medium was added and incubation was continued for 48 hrs. Cells were harvested and assessed for viral RNA quantification by Real Time PCR. To analyze the effect of siRNA on HCV infection, serum infected Huh-7 cells were again seeded after three days of infection in 24-well plates and grown to 80% confluence with 2 ml medium. The cells were transfected with or without 40 nM/well of Core siRNA/shRNA using Lipofectamine™ 2000 (Invitrogen Life technologies, CA) according to the manufacturer's protocol. Cells were harvested and intracellular HCV RNA levels were quantified by Real Time PCR.

### Viral load

Cells were harvested for Intracellular viral RNA determination using Gentra RNA isolation kit (Gentra System Pennsylvania, USA) according to the manufacturer's instructions. For viral quantification Sacace HCV quantitative analysis kit (Sacace Biotechnologies Caserta, Italy) (quantification assay based on the detection of 5'UTR of viral copies) was used by Real Time PCR on cells 3^rd ^day post infection. Briefly, 10 μl of extracted viral RNA was mixed with an internal control derived from 5'UTR provided by Sacace HCV Real TM Quant kit and subjected to viral quantification using Real Time PCR SmartCycler II system (Cepheid Sunnyvale, USA).

### Statistical analysis

All statistical analysis was done using SPSS software (version 16.0, SPSS Inc). Data are presented as mean ± SD. Numerical data were analyzed using student's t-test and ANOVA. P value < 0.05 was considered statistically significant.

## List of abbreviations

E1, E2: Envelop proteins 1, 2; HCC: Hepatocellular carcinoma; HCV: Hepatitis C; PEG-INF-α: pegylated interferon alpha; RNAI: RNA interference; SHRNA: short hairpin RNA; SIRNAS: small interfering RNAs.

## Competing interests

The authors declare that they have no competing interests.

## Authors' contributions

SK, SJ, BI, WA, SA, AP, MK and BS prepared and write manuscript, and perform lab work. SH was the principal investigator and provides all facilitates to complete this work. All authors read and approved final manuscript.

## Authors' information

Saba Khaliq (MSc Zoology) and Shah Jahan (BS Hons) are both PhD scholars, Bushra Ijaz (M Phil Molecular Biology) and Waqar Ahmad (M Phil Chemistry) are Research Officer, Sultan Asad, Asim Pervaiz, Baila Samreen and Mahwish Khan are M Phil scholars, while Sajida Hassan (PhD Molecular Biology) is Principal Investigator at CEMB, University of the Punjab, Lahore
